# Hierarchy Establishment in Growing Finishing Pigs: Impacts on Behavior, Growth Performance, and Physiological Parameters

**DOI:** 10.3390/ani13020292

**Published:** 2023-01-14

**Authors:** Angela Cristina da Fonseca de Oliveira, Saulo Henrique Webber, Yuliaxis Ramayo-Caldas, Antoni Dalmau, Leandro Batista Costa

**Affiliations:** 1 Graduate Program of Animal Science, Pontifícia Universidade Católica do Paraná-PUCPR, Curitiba 80215-901, Brazil; 2 Institut de Recerca i Tecnologia Agroalimentàries-IRTA, Porcine Control and Evaluation, 17121 Monells, Spain

**Keywords:** agonistic contact, coping style, dominance sociomatrix, social ranking, swine

## Abstract

**Simple Summary:**

Pigs are social animals that live in groups with well-established social structures and have always been a key species in debates over farm animal welfare. However, intensive production systems often fail to adequately consider pigs’ social needs. Group housing still presents major welfare concerns due to increased aggression for up to 48 h after pigs are mixed, with potentially chronic levels of aggression if stable social groups are not established. In our study we analyzed pig behavior, performance and physiological parameters after repeated mixing events. We compared individuals, using a dominance sociomatrix, and tested different hypothesis concerning pigs occupying different social ranking. The results suggest that hierarchical classification influenced feeding behavior and that pigs developed a possible compensation skill. Our findings highlight the importance of understanding the role of hierarchy and its biological relevance in group-housed pigs. Having a better understanding of individual differences, according to their social rank, may help producers and researchers identify and implement management strategies to reduce agonistic interactions and promote affiliative behaviors.

**Abstract:**

In recent years, an increased number of studies have dealt with the analysis of social dominance related to animal behavior, physiology, and performance. This study aimed to investigate whether hierarchical ranking affects the coping style, non-social behavior during open field and novel object tests, performance, and physiological parameters of pigs. A total of 48 growing pigs (24 barrows and 24 females) were mixed three times during the growing–finishing period. The social and non-social behaviors of pigs were directly noted, and three behavioral tests were performed during the experimental period. Performance and physiological parameters were also recorded. Statistical analysis considered hierarchical classification (dominant vs. intermediary vs. subordinate) and *p*-values ≤ 0.05 were considered significant. After three regroupings, the pigs in different hierarchical classifications showed no change in hair cortisol values and open-field and novel object tests. Mean corpuscular hemoglobin concentration and leukocyte values increased in intermediary pigs, and the lowest counts were found in pigs classified as dominants. Furthermore, dominant pigs visited the feeder more but spent shorter time there compared to subordinate and intermediary pigs. Our results suggest that hierarchical classification influenced feeding behavior and physiological parameters without affecting cortisol values and growth performance, demonstrating a possible compensation skill.

## 1. Introduction

Dominance hierarchy, a system whereby animals of high rank exhibit superiority in a competitive situation over those of lower rank, is a common form of group organization amongst the vertebrates [1]. The importance and prevalence of dominance hierarchies in nature [2] led to the development of methods to evaluate the establishment of social ranking [3,4]. The stress resulting during rank establishment and position changes strongly influences the behavior and neuroendocrine processes of animals (i.e., when an animal loses its position) [5]. Therefore, the social status of an animal is an important factor that can be used to determine its response to social stress [6].

Previous research investigated the effects of regrouping on the social status of pigs [7,8,9], and the results revealed that large individual differences occurred in response to social stress, indicating that coping styles are different among animals of different social rank [10]. Coping style is defined as the consistent differences in the behavioral and physiological responses of individuals to stressors [11]. Consistent individual differences are thought to be a mechanism that organisms use to adapt to their environment [12,13]. In the proactive versus reactive pig type hypothesis, proactive animals tend to be more aggressive, bold, and rigid in their behavioral responses, whereas reactive animals tend to be more shy, passive, and flexible [11].

The use of behavioral tests to evaluate animal traits is one of the most common method of assessing individual differences in captive animals [14,15]. The backtest is a method adapted from the tonic immobility test in chickens, and it is frequently used to measure coping style, in which pigs are classified as proactive (high-resisting pigs) or reactive (low-resisting pigs) [16]. Behavior tests such as the novel environment test (i.e., open field test) and novel object test were originally designed to measure curiosity in rats (novel object test [17]) and emotionality in rats and mice [18]. More recently, novel environment and object tests have been used to measure exploration and fearfulness in a variety of species [19]. Due to welfare concerns in pig production, efforts to reduce social aggression and resulting damages have mainly focused on agonistic interactions [20]. Having a better understanding of individual pig behavior, performance, and physiological differences, according to their social rank, may help producers and researchers identify and implement management strategies to reduce agonistic interactions and promote affiliative behaviors [20].

The hypothesis of the present study is that dominant pigs will show proactive behavior during the backtest with a high number of escape attempts. Another hypothesis is that dominant pigs will show high quadrant occupation during open field tests, and during novel object tests, and they will show low latency to reach an object and spend more time manipulating it. Furthermore, due to the better control of resources by dominant animals [1], it is also expected that high-ranking individuals will show better performance and blood parameters (neutrophil/lymphocyte ratio) and low hair cortisol levels compared to those low in the hierarchy [6,7,8,9]. Therefore, the objective of the present study was to determine whether hierarchical ranking (dominant vs. intermediary vs. subordinate) affects the copying style, non-social behavior during open field and novel object tests (locomotion, exploration, defecate and urinate behaviors), performance, hair cortisol levels, and blood parameters of pigs.

## 2. Materials and Methods

The experimental design was divided into two phases for data collection: 1. Nursery phase and 2. Growing-finishing phase. The first phase comprised the adaptation period, and it lasted for 30 days; the activities were as follows: arrival of piglets at the institute’s facilities (IRTA, Monells, Spain—41°58′34.02″ S, 2°59′51.35″ W); weighing and identification of piglets; collection of initial blood samples; and record of backtest data (details below). The second phase comprised the experimental period, and it lasted for 127 days; the activities were as follows: reallocation of pigs to the growing-finishing facilities; implementation of electronic feed stations; beginning of the experimental treatment; behavioral tests (open field and novel object); collection of hair samples (cortisol analysis); and collection of final blood samples. The housing conditions and management procedures followed those of the EU pig standards—Council Directive 2008/120/EC of 18 December 2008. Enrichment material (chains and wood fixed to the wall) was made available in each pen during all project phases (nursery phase and growing-finishing phase). This research adhered to the legal requirements of the country, and the study was carried out according to all institutional guidelines. The procedures used were approved by the Comisión de Experimentación Animal de La Generalitat de Catalunya (protocol number —10329).

### 2.1. Nursery Phase

A total of 48 weaned piglets (Duroc Commercial Line—24 barrows and 24 females), with an initial average body weight (BW) of 7.95 ± 1.47 kg were allocated at the institute’s nursery facilities and distributed into four pens (climate-controlled room with twelve animals per pen—12.5 m^2^). The pens included a fully slatted floor, semi-automatic trough, and nipple drinker. Feed and water were provided ad libitum. The ambient temperature was set to 24 °C, and the light regime was set to a 12 h light-dark cycle. After arrival, the piglets were weighed and identified. The identification was carried out using two ear tags. The first tag contained the number for general identification (electronic feeding stations chip to growing-finishing phase) and another one was used during continuous observation tests (six colors of tags: yellow, green, blue, lilac, white, and orange). To differentiate gender (male vs. females), a blue spray was applied on the dorsal region of the barrow.

#### 2.1.1. Blood Samples

To evaluate the physiological parameters of the pigs, blood samples were collected at the beginning (nursery period—day 27) and end of the experimental period (day 127). All the animals were sampled. The pigs were restrained by the snout using a loop, and blood samples were obtained from the jugular vein. The animal was maintained in a standing position and its head at a 30-degree angle. Thereafter, 8 mL of blood was collected and aseptically stored in tubes containing the anticoagulant ethylenediaminetetraacetic acid (EDTA, Becton Dickinson, USA) for analysis of blood count (hematocrit, hemoglobin, hematies, mean corpuscular volume, platelets, leukocytes, eosinophils, basophils, lymphocytes, monocytes, neutrophils, and neutrophil/lymphocyte ratio).

#### 2.1.2. Backtest

**Figure 1:**
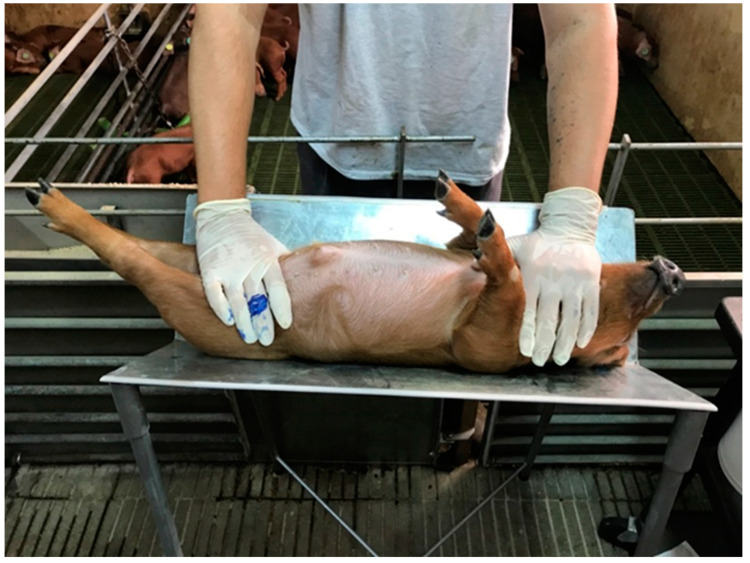
Piglet subjected to laid supine during backtest.

Four days after the pigs arrived at the institute (nursery period—day 4), they were subjected to a backtest (38 ± 2 days of age) and classified as described below. The test was carried out in the corridor near the home pen. Based on preliminary studies, a V-shaped cradle was developed [21]. The structure was made of an uncoated metal with a 120-degree angle and fixed to a support with four columns (Figure 1). The piglet was laid supine for 60 s and restrained by placing one hand gently on the neck and using the other hand to extend and support the hind legs.

The total number of escape attempts, duration of events, and total number of vocalizations were recorded [21]. In the present study, any movement by the piglet to return to the stationary position was considered an escape attempt. Each bout of struggling with at least one of the hind legs was counted as an escape attempt. A bout was terminated when the piglet stopped struggling or paused by stretching or relaxing its legs. If a pig made an escape attempt at the end of the trial, the period was extended until the end of the attempt [22]. Pigs were classified based on the number of escape attempts displayed during the test. The number of escape attempts was indicative of the overall reaction pattern of pigs in the backtest, as it is generally related to the total duration of escape behavior [22,23,24], latency to the first escape attempt [22,23] and number of vocalizations [22,23,24] in this test. A pig was classified as proactive/high-resisting (HR) if it performed more than two escape attempts and reactive/low-resisting (LR) if it performed fewer than two escape attempts [21].

### 2.2. Growing–Finishing Phase

In the second phase of the project, the 48 pigs were moved from nursery pens to the growing facilities of the institute. The pigs were distributed into four pens according to weight (pen from the lightest to the heaviest animals). The experimental unit consisted of 12 pigs (six barrows and six females), with an initial average body weight (BW) of 18.63 ± 3.05 kg and a final average BW of 129.98 ± 10.04 kg. The experimental protocol lasted for 127 days. The growing facilities included pens with a fully slatted floor (5 m × 2.6 m), an electronic feeder system, and a nipple drinker. The unobstructed floor area available to each rearing pig was > 1 m^2^. The room was climate-controlled, and the temperature was set to 19 ± 2 °C with a light regime of 12 h light-dark cycle. Throughout the experimental period the pigs received feed and water ad libitum. The feed was an isonutritive diet, formulated according to the nutrient requirements for each respective period [25].

#### 2.2.1. Social Stress

**Figure 2:**
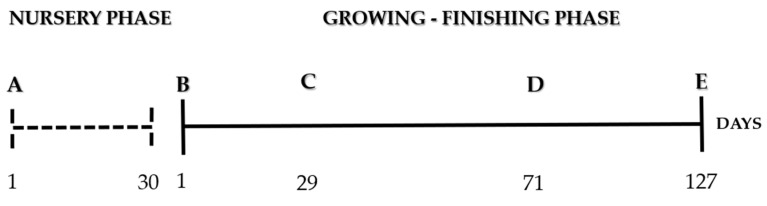
Experiment timeline. This study was conducted for 157 days according to this timeline sequence: A: nursery phase (30 days)—arrival of piglets at the institute facilities and adaptation period; B: growing–finishing phase (127 day)—beginning of the experimental period and first mix performed; C: female mix (second mix performed); D: barrow mix (third mix performed); E: end of the experimental period and pig slaughter.

After pigs were changed from nursery to growth facilities (first mix—day 1), they were mixed two more times during experimental period (Figure 2).

To generate social stress by promoting a resident vs. intruder scenario among the pigs, the mixes were performed in groups and the criteria chosen was gender, since the stalls had six males and six females each. The second mix was in the fifth week of the experiment (day 29), and only the females switched places. As all pens accommodated six barrows and six females, resident barrows remained unchanged. All females from pen number 3 switched places with all females from pen number 6. Conversely, all females from pen number 4 switched places with females from pen number 5. The third mix was for the barrows and was in the eleventh week of the study (day 71). All barrows from pen number 3 switched places with all barrows from pen number 5, and all barrows from pen number 4 switched places with all barrows from pen number 6. Resident females remained unchanged. The study was divided into three periods for results analysis according to the mix performed: period I—first mix performed: start of growing–finishing phase until the female mix (lasted 28 d); period II—second mix performed: start with female mix until the barrow mix (lasted 42 d); period III—third mix performed: start with barrow mix until the end of the experimental protocol (lasted 57 d).

#### 2.2.2. Sampling Methods after Mixing

The sampling methods applied in the present study were adapted from [26] and the behavior aspects were according to [27]. Based on the standard time of dominance establishment [28,29], the groups were directly observed during the first 72 h after mixing. All observations were performed by two previously trained observers during the entire experimental period to avoid the bias effect of observers. The animals were already familiar with the presence of the observers since they had been managing the pens during the nursery phase (period of pigs’ adaptation). During observations, the evaluators were positioned in front of the pen at the corridor of the shed to directly record. During the entire study the same type of clothes (jumpsuit) with the same colors (gray) were used, to avoid possible influences on the behavior of the animals.

Based on continuous event sampling, all occurrences of agonistic interaction (AI) were recorded for 3 min [26] (Table 1). In the present study, an agonistic interaction was considered when an aggressive behavior was delivered by one pig towards another and lasted for more than 1 s. If one animal showed submissive behavior and the two opponents were separated for at least 5 s, the event was considered finished [30,31]. For each agonistic interaction, the author, receiver, winner, loser, and the pen area where the interaction occurred were recorded. Only agonistic interactions between two animals with a clear author and receiver and a clear winner and loser were used for further analyses. The event was defined as indecisive if clear submissive behavior was not detected.

After assessing the AI (3 min), an instantaneous sampling (scan sampling) was carried out to record the main activities of the animals (1 min) [26]. The behaviors recorded were eating, exploration of the ambient environment, exploration of the environmental enrichment material (chains and wood fixed to the wall), lying (on sternum or laterally), drinking, positive and negative interactions, and others (any behavior that does not fit into the activities described above). Positive interactions were defined as sniffing, nosing, licking, and moving away gently from the animal without aggressive or flight reaction from the individual.

These evaluations were conducted for a total of two consecutive hours in the morning (0900 to 1100 h) and two consecutive hours in the afternoon (1400 to 1600 h). A total of 12 continuous event sampling (assessing of agonistic interactions) and 12 scan sampling per pen/day were completed. At the end of three days, each pen was evaluated 36 times for continuous event sampling and 36 times for scan sampling, totaling 288 observations per period.

#### 2.2.3. Behavioral Tests

**Figure 3:**
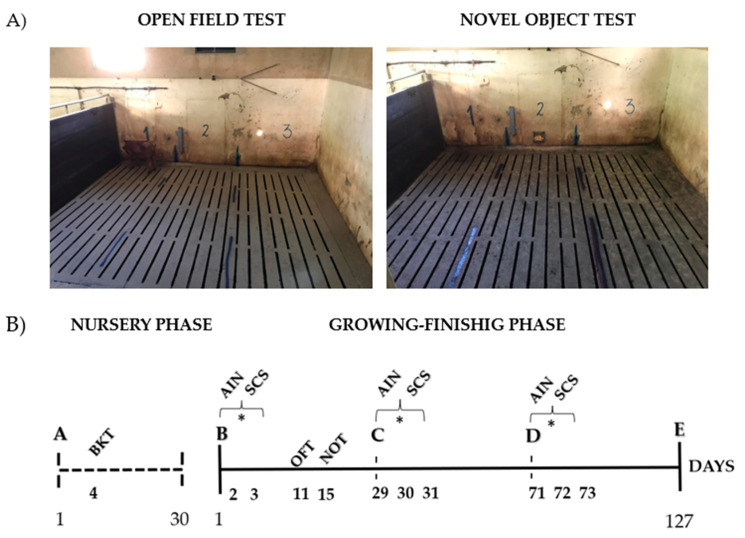
(**A**) Record of the pen area used for tests and (**B**) the experiment timeline in days, with all behavioral assessments. Symbols correspondence: A: nursery phase (30 days)—arrival of pigs at the institute facilities and adaptation period; B: growing-finishing phase (127 days)—beginning of the experimental period and first mix performed; C: female mix (second mix performed); D: barrow mix (third mix performed); E: end of the experimental period and pig slaughter; BKT—backtest; AIN—assessment of agonistic interactions by continuous event sampling; SCS—scan sampling; OFT—open field test; NOT—novel object test; * Measures performed during 72 h after mixing.

The test pen designed for open field (OFT) and novel object test (NOT) was located inside the shed (5.0 m × 2.6 m), with a fully concrete floor. All the sides were covered with a black plastic material to block the view of the animals. Figure 3 shows A) a test pen record and B) the experiment timeline in days. All the behavioral assessments performed (continuous event sampling and behavioral tests) were based on this period. The OFT and NOT were carried out in the morning (0800 to 1100 h) and afternoon (1300 to 1600 h), and the animals were randomly chosen. All enrolled pigs were evaluated (see details below). The behaviors were directly noted by two observers who had been previously trained to ensure consistency in the results (inter-observer reliability). The ethogram used was adapted from [33,34,35] and each measure was clearly defined as below (Table 2). The OFT was performed once during the study in Period—I. The animals were individually observed in the test arena, and the measures were recorded within 2 min to consider reactivity, quadrant occupation, vocalization, and urinate and defecate behaviors. For the evaluation of reactivity, three behavioral parameters were used: activity, exploration, and escape attempts. The NOT was performed once during the study in Period—I. Previous studies have examined porcine flavor preferences [4,36] and found that sweet flavoring agents have been used to create interest in solid food. In this test, the novel object was represented by a feeder with chopped apples. The animals were individually observed in the test arena, and the measures were recorded within 2 min: reactivity (activity, exploration and escape attempts), quadrants occupation, vocalization, urinate and defecate behaviors, and novel object manipulation. At the end of each test, the arena was briefly cleaned. 

#### 2.2.4. Growth Performance and Feeding Behavior

The number of visits to the feeder, length of the event (meal duration—s), and total feed consumption per event (kg) were recorded daily through electronic feeding stations. Once a month each animal was individually weighed, for a total of four times during the experimental period. Based on these data, the initial and final BW, average daily feed intake (ADFI), average daily gain (ADG), and feed conversion ratio (F:G) were determined.

#### 2.2.5. Hair Cortisol

The authors opted for hair cortisol analysis, instead of blood or feces samples, because hair cortisol is a non-invasive means that captures long-term cortisol secretion [37]. The hair samples were collected once at the end of the experimental period (day 127). The region intended for hair collection was shaved at the beginning of the study after the initial blood samples had been collected (nursery period—day 27). Thus, the hair collected at the end of the study represented only the period that the animals were under experimentation. The samples were obtained from the dorso-lumbar (L) region by taking advantage of the restraint provided during the regular weighing. Pigs were gently accompanied to the weighing scales, which had a two-door system for access and exit. Hair was collected by shaving close to the skin with clippers, without removing the root of the hair and ensuring that the hair follicle was not in the sample. After sampling, the hair was stored at an ambient temperature inside a hermetically sealed bag until analyzed. Cortisol extraction was conducted following the method of [38].

#### 2.2.6. Statistical Analysis and Dominance Sociomatrix

**Figure 4:**
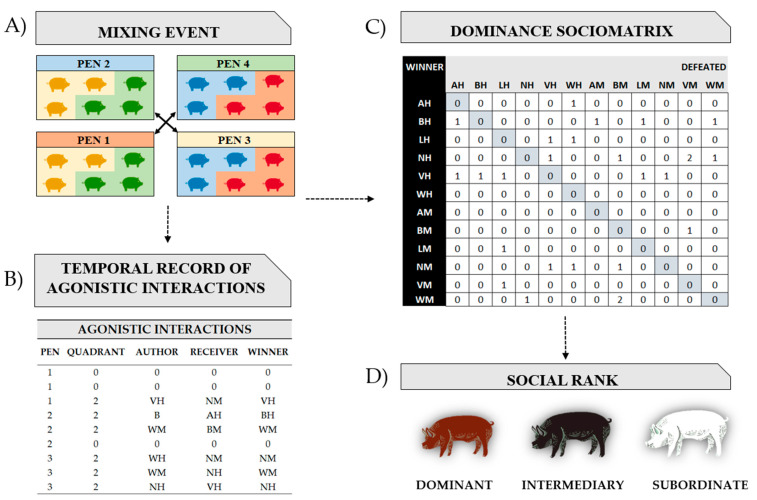
Diagram highlighting the different steps required to establish dominance hierarchies. The outcome of dyadic agonistic interactions between individuals was recorded in the temporal sequence of winners and losers in a matrix. (**A**) mixing event; (**B**) Continuous event sampling recording all occurrences of agonistic interactions [72 h after mixing]; (**C**) Accumulated frequencies of wins and losses per pen for each individual; (**D**) Social rank establishment: dominant (higher number of victories and lower number of defeats), subordinate (higher number of defeats and lower number of victories), and intermediary (low number of interactions within the pen).

For statistical analysis, pigs had been previously classified as dominant, intermediary, or subordinate. The accumulated frequencies of wins and losses per pen for each individual were aggregated into a separate win/loss frequency sociomatrix, in which the winners were listed in rows and losers in columns (Figure 4). Using the method in a previous study reported by [4,36], in each cell of the matrix, “1” was assigned to pigs in rows who won more often against pigs in columns. If the fight was a tie, both pigs received “0”. The individuals were classified as: 1. dominant (higher number of victories and lower number of defeats), 2. subordinate (higher number of defeats and lower number of victories), and 3. intermediary (low number of interactions within the pen). A sociomatrix was created per pen for each mix performed. The animals were divided into four pens, and three mixes were performed during the study, implying that 12 matrices were performed.

The growth performance, physiological, and behavioral data were grouped according to the hierarchical classification of the animals (dominant vs. intermediary vs. subordinate). Statistical analyses were performed using STATGRAPHICS Centurion XVI statistical Software ^®^, Version 16.11. (2022 Statgraphics Technologies, Inc. The Plains, VA, USA) Data from parametric variables were compared using analysis of variance (ANOVA-Type III) followed by a Tukey’s test when homogeneity of variance was observed (Levene’s test). Where the data did not show normality (Shapiro–Wilk) or homogeneity of variance (Levene’s test), a Wilcoxon test was conducted. The results are presented as the mean ± standard error. Two statistical analyses were used for non-parametric data, depending on the type of data obtained. Results from quadrant occupation and number of vocalizations were compared using a Mann–Whitney (Wilcoxon) W-test. Nonparametric data from binary analyzes (yes/no) were correlated using Kendall’s tau-b (τb) correlation coefficient. Non-parametric data are presented as the median (minimum–maximum) values and the description of each test used is indicated at the footnote of the table. *p*-values ≤ 0.05 were considered significant.

## 3. Results

### 3.1. Physiological Measures

The means (±SE) of complete blood counts (CBC) and white blood cells (WBC) are present in Table 3. For initial values, no significant statistical differences were found for CBC parameters. For WBC, the value of monocytes was higher in the subordinate group than in intermediary and dominant groups (*p* = 0.056). For the final values, the highest MCHC and leukocytes count were found in the intermediary group (*p* = 0.040), and they were statistically different from those in the dominant group, which were statistically equal to those in subordinate group. In addition, the initial and final values of monocyte counts varied between the groups; the intermediary group had the highest values compared to those in other groups (*p* = 0.058).

### 3.2. Backtest

The means (± SE) and medians (minimum–maximum) of the backtests are present in Table 4. The latency (time until the first escape attempt) and urinate variables were not included in the Table, because all the piglets had a latency below 1 s and did not urinate during the test. No statistical difference was observed between the duration of escape attempts and number of vocalizations. The number of vocalizations per piglet ranged from 1 to 3 grunts. Furthermore, all the piglets needed only one attempt to get out of the supine position—reactivity variable (*n*). For the defecate variable, values did not differ statistically between groups.

### 3.3. Performance and Feeding Behavior

The means (± SE) and medians (minimum–maximum) of growth performance and feeding behavior, respectively, according to period and dominance sociomatrix classification, are presented in Table 5. No difference was found during period—I. During period—II, the intermediary group spent more time at the feeder (ATES) than the dominant group did (*p* = 0.053). Pigs classified as subordinates showed the same values for ATES compared to the intermediary and dominant groups (*p* > 0.05). During period—III, pigs of dominant group made the highest number of visits to the feeder (NVF) compared to the other groups.

### 3.4. Open Field and Novel Object Test

Data from the open field test (OFT) and novel object test (NOT) are presented in Table 6 and Table 7, respectively. For OFT, the groups presented the same values of activity in the test arena and number of escape attempts (reactivity). The pigs also occupied the quadrants in the arena and vocalized at the same frequency (*p* > 0.05). Regarding defecate behavior, no difference was observed between the numbers of pigs that defecated during the test. No pig in the dominant group urinated during the OFT (*p* < 0.05), whereas the pigs classified as intermediary and subordinate showed statistically equal values.

For NOT, no difference was found between the variables analyzed. The pigs showed close latency values until they reached the feeder and when handling the apples (*p* > 0.05). The same pattern was observed for the quadrant occupation, number of vocalizations, escape attempts, and defecate and urinate behaviors.

## 4. Discussion

### 4.1. Performance and Physiological Parameter

According to the results obtained, some changes were found in the blood counts and feeding behavior. The mean corpuscular hemoglobin concentration (MCHC) and leukocytes values were high in the intermediary group, and the lowest counts were found in the dominant group. The animals in the subordinate group showed intermediate values for both variables. Furthermore, the intermediary group also had the highest monocyte count (Mon). Hematological profile data for pig commercial lines are scarce, despite the increasing interest of this approach in research [39]. The pig erythrocyte is highly susceptible to hemolysis by hypotonic saline and is more fragile than that of other species; this explains the MCHC values. Furthermore, leukocytosis is a common reaction in pigs to stress or infection. Similar to other animals, pigs are easily stressed during handling and restraint and their stress response develops within 2 min, rapidly affecting the leukogram [40]. Catecholamines, which are usually produced at the beginning of stress or during a short-term stressful stimulus, causes leukocytes to increase in their systemic circulation [41]. Leukocyte and monocyte count recorded in this study can be considered to be within the normal range for hematological parameters as reported by [42] and [39], indicating that the observed fluctuations did not exceed the threshold considered pathological. Previous studies that showed differences in hematological parameters and cortisol collected samples immediately after the stress factor was applied, within a short period after mixing [43,44]. Furthermore, according to Coutellier et al. [45], the acute response to mixing decreases over time and repeated regrouping, suggesting a habituation to the stressful situation. Therefore, the influence of a chronic intermittent stressor on hematological parameters and cortisol may have may have been diluted over the study period. Furthermore, despite its wide use, cortisol concentration is influenced by many factors that could limit its use as a stress biomarker [46,47], which may explain the low variation between groups. The average concentration of cortisol in pigs decrease with age, reaching a stable profile around 20 weeks of age [48,49], and gender, with concentration in males being approximately 15% higher than that in females [49]. All these factors linked to cortisol as a biomarker may have contributed to equalize the samples between treatments, thereby leading to the absence of statistical significance.

Regarding performance parameters and feeding behavior, the animals classified as dominant visited the feeder more during the experimental period (period—III) but spent less time in the feeder (period—II). However, the ADFI in these periods did not change between groups, indicating that although pigs went more frequently to the feeder and spent shorter time at it, they were able to balance the consumption of animals hierarchically below. The same pattern of high number of visits and low length of time was also observed by [50] and [51] in pigs classified as dominant, and similar to the present study, there was no statistical difference in the other performance parameters and growth rate of the pigs. According to the review presented by [52], there are two theories on feed competition and how group housing influences feeding patterns. The first one explains that pigs adapt their feeding patterns in response to competition for access to the feed. The second one, linked to the first, suggest that social facilitation enhances feed competition and as a consequence, alters feeding patterns. If displacement is difficult, that is, when a protective crate is used [53], pigs are forced to withdraw from the feeder less often but may have more difficulty in accessing the feeder, leading to a low feeding frequency and high feeding duration per visit during competition. If displacement is easy, pigs, especially small ones [54], are forced to withdraw often and will hence maintain their intake by visiting the feeder often with small visits time at each time. In the present study, the electronic feeders did not present any lateral protection, hence, the interruption by other animals was facilitated. Furthermore, as mentioned in the methodology, the blocking of animals was performed according to weight. Thus, there was no discrepancy between the body sizes of the individuals within the pen. According to [55], pigs of similar weight have more trouble establishing a clear dominance order. Moreover, agonistic acts are less frequent at the end of fattening [56] but still exist because of permanent attempts by pigs of close hierarchical ranks to improve their position [57,58]. Thus, the findings in the present study can be explained by the number of agonistic interactions, in which the dominant animals were involved, either by facilitated displacement or by the attempts of other animals to improve their position in the hierarchical rank. In addition, dominant animals were considered to be the one that won more fights, but it is possible that an animal more involved in fights may be positively biased in this classification, and it is not clear whether a truly dominant animal would be so involved in fights. A constant analysis of the number and type of social interactions, throughout the grower and finisher period, needs to be conducted to better elucidate the hypothesis raised in this study.

### 4.2. Backtest, OFT, and NOT

Over the last 27 years, studies on pig behavior have investigated links between coping style, physiological responses, social and non-social traits, and these have major implications for livestock [59,60]. Pigs with reactive coping styles were more influenced by their housing environment than proactive pigs. In turn, housing influenced the immune response, social behavior, and judgment tasks of reactive pigs, and this has serious implications on the management of pigs [61]. As mentioned above, one of the objectives of the present work was to investigate the impacts of hierarchical classification (dominant vs. subordinate vs. intermediary pigs) on the coping style of pigs. The hypothesis was that pigs from different hierarchical ranks would present different levels of shyness-boldness, exploration (measured in a novel situation), and activity. However, pigs showed little variation based on the results observed for the backtest, OFT, and FMT.

In a review carried out by O’Malley et al. [61], the most popular behavioral test applied was the backtest, which was used in 67.5% of 83 studies. Hessing introduced the backtest in young piglets in the early 1990s and hypothesized that it could detect coping strategies [22], but the inconsistent and ambiguous results seen across studies may be due to variation in methodology or probably because the backtest is an inappropriate method for measuring personality traits in pigs [62]. A possible explanation for the low variation found in the present data was the age of the pigs at which the test was performed. Previous studies evaluated the animals between 5 and 26 days of age [21,22,23,24,25,26,27,28,29,30,31,32,33,34,35,36,37,38,39,40,41,42,43,44,45,46,47,48,49,50,51,52,53,54,55,56,57,58,59,60,61,62,63]. In the present study, piglets were evaluated within 38 ± 2 days of age. Since the animals were older, body size may have facilitated escape movements. All animals were able to turn around with just one attempt, and the duration of the attempt ranged from 1 to 3 s for all animals.

Regarding the OFT and NOT, no significant differences were found in most of the variables observed. The only significant difference was between the number of pigs that urinated during the OFT; it was higher for intermediary animals compared to that for dominant ones. These tests are often used in pigs [64] and the variables most commonly recorded are locomotor activity (lying, standing, and exploration), defecate or urinate behaviors, vocalizations (squeals, grunts), latency (time spent to consume the food) and duration (time spent manipulating the food). As mentioned by [64], social motivation can be the primary factor affecting open-field behavior, especially when group-reared animals are tested individually. Since the pigs were classified based on group assessments (social interactions) and the animals were tested individually in OFT and FMT, the absence of social motivation may dilute the effect of the hierarchical ranking. According to the data collected, there were few studies that investigated differences between coping style, hierarchical ranking, and performance and physiological parameters. Unfortunately, different methodologies present numerous challenges for animal researchers [61].

## 5. Conclusions

After three regroupings during the growing-finishing period, pigs in different hierarchical classifications showed no differences in hair cortisol values and open-field and novel object tests. The blood count and white blood cell analysis showed variation between groups. Furthermore, compared to pigs of other hierarchical ranks, the dominant pigs visited the feeder much more but spent shorter time there. Our results suggest that hierarchical classification influenced feeding behavior and physiological parameters, without affecting cortisol values and growth performance, demonstrating a possible compensation skill. Research on pig coping style and hierarchical establishment currently has issues that prevent the application of this information to make realistic management recommendations. Future studies are needed to develop tests that are ecologically relevant to pigs, and that can consistently be applied across studies.

## Figures and Tables

**Table 1:** Definition of the ethological patterns used.

Term	* Definition
Agonistic interaction	Physical contact between individuals with or without reaction on the part of the receiver, leading to an attack (unilateral action—the receiver does not bite back) or a fight (bilateral action—the receiver counter-attacks)
Submissive behavior	Body-turning (receiver pig turns whole body 180 degrees to protect head and ear) and is usually accompanied by an ear bite and flight response of the attacked pig
Author	Attacking pig
Receiver	Pig being attacked
Winner	A pig is considered a winner when the other individual involved in the agonistic interaction shows a flight and/or submissive behavior. The winner may be the individual who started the agonistic interaction or not
Loser	The loser of an agonistic interaction is the one who showed an escape movement during the event. The loser may be the individual who initiated the agonistic interaction or not

* The ethogram applied in the present study was adapted from [32].

**Table 2:** Description of the behavioral measures for open field and novel object tests performed during the experimental period.

Measure		Description
Reactivity	Activity	-No movement of any portion of the pig’s body was visible for a period > 2 s. The duration includes the beginning of the inactivity until any movement of the body.
		-No movement of any portion of the pig’s body was visible during the entire testing period (freezing).
	Exploration	-Time it takes to move at least three feet (seconds/minute).
		-Time spent manipulating/exploring the floor or walls with its nose (seconds).
	Escape attempts	-Number of times the animal raised its front legs against the wall (seconds).
Quadrant occupation		-Number of times the animals crossed the line of a quadrant with both front legs.
Vocalization		-Number of vocalizations (squeals and grunts).
Eliminatory Conducts		-Urinate and defecate (yes/no).
Novel object manipulation *		-Latency to touch the feeder with apples (seconds).
		-Time spent manipulating/touching the apples (seconds).

* Novel object manipulation: measured applied only during novel object test.

**Table 3:** Initial and final average blood count (mean ± SE) and cortisol samples (mean ± SE) of growing and finishing pigs based on dominance sociomatrix classification.

		Initial Values				Final Values			
^1^ Item	-	SUB	INT	DOM	*p*-Value	SUB	INT	DOM	*p*-Value
CBC—Complete Blood Count									
HTC	%	37.66 ± 0.96	36.36 ± 0.69	36.88 ± 1.05	0.551	42.02 ± 1.32	42.11 ± 0.96	42.32 ± 1.32	0.987
HMB	g/dL	10.42 ± 0.29	10.35 ± 0.16	10.44 ± 0.24	0.932	12.85 ± 0.35	13.14 ± 0.25	12.77 ± 0.35	0.641
HMT (10^6^)	/µL	6.31 ± 0.12	6.10 ± 0.09	6.38 ± 0.13	0.166	7.33 ± 0.23	7.19 ± 0.17	6.96 ± 0.23	0.521
MCV	fL	59.76 ± 1.33	59.67 ± 0.96	57.94 ± 1.46	0.574	57.22 ± 1.48	58.67 ± 1.07	61.57 ± 1.48	0.114
MCH	pg	16.53 ± 0.29	17.00 ± 0.21	16.38 ± 0.32	0.204	17.55 ± 0.41	18.31 ± 0.29	18.58 ± 0.41	0.180
MCHC	g/dL	27.76 ± 0.52	28.60 ± 0.38	28.31 ± 0.57	0.434	30.72 ± 0.31 ^AB^	31.22 ± 0.22 ^A^	30.24 ± 0.31 ^B^	0.040
PLT (10^5^)	/µL	3.87 ± 0.55	3.78 ± 0.37	4.04 ± 0.58	0.932	3.00 ± 0.34	2.40 ± 0.24	2.72 ± 0.34	0.330
WBC—White Blood Cells									
Lkc (10^3^)	%	15.61 ± 0.08	14.73 ± 0.06	17.42 ± 0.09	0.036	16.58 ± 0.11 ^AB^	18.21 ± 0.08 ^A^	14.53 ± 0.1 ^B^	0.044
Eos (10^2^)	/µL	5.86 ± 1.17	3.61 ± 0.08	4.34 ± 1.23	0.311	6.18 ± 1.20	6.57 ± 0.83	5.13 ± 1.15	0.598
Lynf (10^3^)	/µL	5.70 ± 0.58	5.39 ± 0.42	7.05 ± 0.63	0.098	7.86 ± 1.11	9.63 ± 0.80	6.60 ± 1.11	0.087
Mon (10^2^)	/µL	7.48 ± 1.42 ^a^	3.36 ± 1.03 ^b^	3.26 ± 1.58 ^b^	0.056	2.73 ± 1.16 ^B^	6.33 ± 1.16 ^A^	2.42 ± 1.46 ^B^	0.058
Seg (10^3^)	/µL	8.77 ± 0.60	8.71 ± 0.44	9.79 ± 0.67	0.379	7.81 ± 0.52	7.59 ± 0.38	7.26 ± 0.52	0.758
Seg:Lynf	-	1.61 ± 0.20	1.77 ± 0.14	1.61 ± 0.22	0.741	1.03 ± 1.12	0.96 ± 0.08	1.20 ± 0.12	0.260
Cortisol	µg	-	-	-	-	0.05 ± 0.002	0.05 ± 0.001	0.05 ± 0.002	0.847

SUB: subordinate; INT: intermediary; DOM: dominant. Period of 127 d under control. ^1^ HTC: Hematocrit; HMB: Hemoglobin; HMT: Hematies; MCV: Mean corpuscular volume; MCH: Mean corpuscular hemoglobin; MCHC: Mean corpuscular hemoglobin concentration; PLT: Platelets; Lkc: leucocytes; Eos: Eosinophils; Lynf: Lymphocytes; Mon: Monocytes; Seg: neutrophils; Seg:lymf: neutrophil/lymphocyte ratio. ^a,b^ Different letters on the same row for hierarchical rank—initial values represent differences between means according to ANOVA followed by Tukey’s test (*p* ≤ 0.05). ^A,B^ Different letters on the same row for hierarchical rank—final values represent differences between means according to ANOVA followed by Tukey’s test (*p* ≤ 0.05).

**Table 4:** Data from backtest according to the dominance sociomatrix classification.

Item	Dominance Sociomatrix			
	SUB	INT	DOM	*p*-Value
^1^ One-Way ANOVA				
Duration (s)	1.16 ± 0.18	1.16 ± 0.13	0.75 ± 0.18	0.142
^2^ Mann–Whitney: W-Test				
Reactivity (n)	1	1	1	-
Vocalization (n)	0 (0–0)	0 (0–1)	0 (0–3)	-
^3^ Kendall’s Tau b				
Defecate (%)	10.42	27.08	10.42	-

SUB: subordinate; INT: intermediary; DOM: dominant. Period of 127 d under control. ^1^ Parametric data are presented as mean ± standard error and compared using ANOVA-Type III followed by Tukey’s test when homogeneity of variance (Levene’s test) was observed. ^2^ Nonparametric data resulting from scores are presented as median (minimum—maximum), and Mann–Whitney (Wilcoxon) W-test was used to compare the medians of the two samples. ^3^ Nonparametric data resulting from binary data (presence/absence) are presented as percentages, and Kendall’s Tau b test was used to compare count and frequency.

**Table 5:** Data of performance (mean ± SE) and feeding behavior (median, min–max) of growing and finishing pigs according to dominance sociomatrix classification and period of mixing.

Item	Period	Dominance Sociomatrix			
		SUB	INT	DOM	*p*-Value
^1^ One-Way ANOVA					
IBW (Kg)	I	25.63 ± 0.94	25.57 ± 0.68	26.92 ± 0.94	0.478
	II	40.72 ± 0.95	41.23 ± 0.69	42.19 ± 0.95	0.542
	III	73.09 ± 1.65	75.64 ± 1.19	75.96 ± 1.65	0.384
FBW (Kg)	I	40.72 ± 0.95	41.23 ± 0.69	42.19 ± 0.95	0.542
	II	73.09 ± 1.65	75.64 ± 1.19	75.96 ± 1.65	0.384
	III	124.20 ± 3.01	131.91 ± 2.17	130.75 ± 3.01	0.118
ADG (Kg)	I	0.79 ± 0.02	0.82 ± 0.02	0.80 ± 0.02	0.530
	II	0.79 ± 0.02	0.84 ± 0.02	0.82 ± 0.02	0.154
	III	0.97 ± 0.04	1.06 ± 0.03	1.03 ± 0.04	0.113
ADFI (Kg)	I	1.56 ± 0.09	1.52 ± 0.07	1.54 ± 0.09	0.942
	II	2.32 ± 0.13	2.50 ± 0.10	2.30 ± 0.13	0.366
	III	2.94 ± 0.15	3.13 ± 0.11	3.27 ± 0.15	0.302
F:G	I	2.01 ± 0.14	1.87 ± 0.10	1.95 ± 0.14	0.726
	II	2.94 ± 0.15	2.98 ± 0.11	2.82 ± 0.15	0.708
	III	3.08 ± 0.13	2.96 ± 0.09	3.15 ± 0.13	0.451
ATES (h)	I	23.28 ± 1.30	25.21 ± 0.94	22.68 ± 1.31	0.240
	II	45.42 ± 3.70 ^ab^	54.51 ± 2.67 ^a^	44.86 ± 3.70 ^b^	0.053
	III	44.97 ± 2.99	50.92 ± 2.16	50.65 ± 2.99	0.250
^2^ Mann–Whitney: W-Test					
NVF (n)	I	396 (245–610)	328 (160–660)	324 (226–718)	-
	II	468.5 (255–719)	535 (189–638)	497.5 (152–916)	-
	III	325.5 (255–676) ^B^	392.0 (201–577) ^B^	437.5 (369–871) ^A^	-

SUB: subordinate; INT: intermediary; DOM: dominant. IBW: initial body weight; FBW: final body weight; ADG: average daily gain; ADFI: average daily feed intake; F:G: feed gain ratio; ATES: average time at the electronic station; NVF: number of visits to the feeder. Period: I—start of experimental control until the mixing of females (28 d); II—mixing of females until the mixing of barrows (42 d); III—mixing of barrows until the end of the experimental protocol (57 d). ^1^ Parametric data are presented as mean ± standard error and compared using ANOVA-Type III followed by Tukey’s test when homogeneity of variance (Levene’s test) was observed. ^2^ Nonparametric data resulting from scores are presented as medians (minimum–maximum), and Mann–Whitney (Wilcoxon) W-test was used to compare the medians of the two samples. ^a,b^ Different letters on the same row for dominance sociomatrix classification represent differences between means according to ANOVA followed by Tukey’s test (*p* ≤ 0.05). ^A,B^ Different letters on the same row for dominance sociomatrix classification represent differences between medians according to Mann–Whitney (Wilcoxon) W-test (*p* ≤ 0.05).

**Table 6:** Data from open field test according to the dominance sociomatrix of finishing pigs.

Item	Dominance Sociomatrix			*p*-Value
	SUB	INT	DOM	
^1^ One-Way ANOVA				
Activity (s)	44.92 ± 6.65	51.58 ± 4.70	64.00 ± 6.65	0.128
^2^ Mann–Whitney: W-Test				
Reactivity (n)	0 (0–0)	0 (0–4)	0 (0–1)	-
QO–1 (n)	3.0 (1–5)	3.0 (0–7)	3.5 (0–11)	-
QO–2 (n)	5.5 (2–9)	6.0 (0–14)	5.5 (2–19)	-
QO–3 (n)	3.0 (0–4)	2.0 (0–6)	3.0 (1–7)	-
Vocalization (n)	7.5 (0–49)	13.5 (0–49)	6.0 (0–93)	-
^3^ Kendall’s Tau b				
Defecate (%)	12.50	37.50	20.83	-
Urinate (%)	6.25 ^ab^	14.58 ^a^	0 ^b^	-

SUB: subordinate; INT: intermediary; DOM: dominant. QO-1: quadrant for animals entering the arena; QO-2: central quadrant; and QO-3: quadrant located laterally to the corral and opposite side of the entrance to the arena. Test arena located inside the shed, with dimensions of 5.0 m × 2.6 m and a fully slatted floor. ^1^ Parametric data are presented as mean ± standard error and compared using ANOVA-Type III followed by Tukey’s test when homogeneity of variance (Levene’s test) was observed. ^2^ Nonparametric data resulting from the scores are presented as medians (minimum–maximum), and Mann–Whitney (Wilcoxon) W-test was used to compare the medians of the two samples. ^3^ Nonparametric data resulting from the binary data (presence/absence) are presented as percentages, and Kendall’s Tau b test was used to compare count and frequency. ^a,b^ Different letters on the same row for dominance sociomatrix classification represent differences between medians according to Kendall’s Tau b test (*p* ≤ 0.05).

**Table 7:** Data from novel object test according to the dominance sociomatrix of finishing pigs.

Item	Dominance Sociomatrix			
	SUB	INT	DOM	*p*-Value
^1^ One-Way ANOVA				
Latency (s)	49.50 ± 13.26	75.52 ± 9.58	54.00 ± 13.26	0.213
Duration (s)	59.08 ± 11.39	30.78 ± 8.22	38.92 ± 11.39	0.142
^2^ Mann–Whitney: W-Test				
Reactivity (n)	0 (0–0)	0 (0–2)	0 (0–1)	-
QO-1 (n)	1 (0–3)	1 (0–4)	1 (0–6)	-
QO-2 (n)	3.5 (1–6)	3 (1–7)	3 (1–8)	-
QO-3 (n)	1.5 (0–4)	2 (0–5)	2 (0–5)	-
Vocalization (n)	7.5 (0–24)	9.0 (0–54)	7.0 (1–22)	-
^3^ Kendall’s Tau b				
Defecate (%)	8.51	25.53	17.02	-
Urinate (%)	2.13	8.51	4.26	-

SUB: subordinate; INT: intermediary; DOM: dominant. QO-1: quadrant for animals entering the arena; QO-2: central quadrant, where the feeders were positioned; and QO-3: quadrant located laterally to the corral and opposite side of the entrance to the arena. Test arena located inside the shed, with dimensions of 5.0 m × 2.6 m and a fully slatted floor. ^1^ Parametric data are presented as mean ± standard error and compared using ANOVA-Type III followed by Tukey’s test when homogeneity of variance (Levene’s test) was observed. ^2^ Nonparametric data resulting from the scores are presented as medians (minimum–maximum), and Mann–Whitney (Wilcoxon) W-test was used to compare the medians of the two samples. ^3^ Nonparametric data resulting from the binary data (presence/absence) are presented as percentages, and Kendall’s Tau b test was used to compare count and frequency.

## Data Availability

The datasets generated during and/or analyzed during the current study are available from the corresponding author on reasonable request.

## References

[1] Meese G.B., Ewbank R. (1973). The establishment and nature of the dominance hierarchy in the domesticated pig. Anim. Behav..

[2] Drews C. (1993). The concept and definition of dominance in animal behavior. Behaviour.

[3] Schmid V.S., de Vries H. (2013). Finding a dominance order most consistent with a linear hierarchy: An improved algorithm for the I&SI method. Anim. Behav..

[4] Tong X., Shen C., Chen R., Gao S., Liu X., Schinckel A.P., Zhou B. (2019). Reestablishment of Social Hierarchies in Weaned Pigs after Mixing. Animals.

[5] Von Holst D. (1998). The concept of stress and its relevance for animal behavior. Adv. Study. Behav..

[6] Fleshner M., Laudenslager M.L., Simons L., Maier S.F. (1989). Reduced serum antibodies associated with social defeat in rats. Physiol. Behav..

[7] de Groot J., Ruis M.A., Scholten J.W., Koolhaas J.M., and Boersma W.J. (2001). Long-term effects of social stress on antiviral immunity in pigs. Physiol. Behav..

[8] Dingemanse N.J., Wolf M. (2013). Between-individual differences in behavioural plasticity within populations: Causes and consequences. Anim. Behav..

[9] Gimsa U., Tuchscherer M., Kanitz E. (2018). Psychosocial Stress and Immunity-What Can We Learn From Pig Studies?. Front. Behav. Neurosci..

[10] Schouten W.G.P., Wiepkema P.R. (1991). Coping styles of tethered sows. Behav. Process..

[11] Koolhaas J.M., Korte S.M., De Boer S.F., Van Der Vegt B.J., Van Reenen C.G., Hopster H., De Jong I.C., Ruis M.A., Blokhuis H.J. (1999). Coping styles in animals: Current status in behavior and stress physiology. Neurosci. Biobehav. Rev..

[12] Sih A., Bell A.M., Johnson J.C., Ziemba R.E. (2004). Behavioral syndromes: An integrative overview. Q. Rev. Biol..

[13] Koolhaas J.M. (2008). Coping style and immunity in animals: Making sense of individual variation. Brain Behav. Immun..

[14] Gosling S. (2001). From mice to men: What can we learn about personality from animal research. Psychol. Bull..

[15] Watters J.V., Powell D.M. (2011). Measuring animal personality for use in population management in zoos: Suggested methods and rationale. Zoo Biol..

[16] Forkman B., Boissy A., Meunier-Salaün M.C., Canali E., Jones R.B. (2007). A critical review of fear tests used on cattle, pigs, sheep, poultry and horses. Physiol. Behav..

[17] Berlyne D.E. (1950). Novelty and curiosity as determinants of exploratory behavior. Br. J. Psychol..

[18] Archer J. (1973). Tests for emotionality in rat and mice: A review. Anim. Behav..

[19] Huang P., Kimball R.T., Mary C.M.S. (2018). Does the use of a multi-trait, multi-test approach to measure animal personality yield different behavioural syndrome results?. Behaviour.

[20] O’Malley C.I., Steibel J.P., Bates R.O., Ernst C.W., Siegford J.M. (2022). The Social Life of Pigs: Changes in affiliative and agonistic behaviors following mixing. Animals.

[21] Zebunke M., Repsilber D., Nürnberg G., Wittenburg D., Puppe B. (2015). The backtest in pigs revisited—An analysis of intra-situational behaviour. Appl. Anim. Behav. Sci..

[22] Hessing M.J.C., Hagelso A.M., van Beek J.A.M., Wiepkema P.R., Schouten W.G.P., Krukow R. (1993). Individual behavioural characteristics in pigs. Appl. Anim. Behav. Sci..

[23] Hessing M.J.C., Hagelso A.M., Schouten W.G.P., Wiepkema P.R., Van Beek J.A.M. (1994). Individual behavioral and physiological strategies in pigs. Physiol. Behav..

[24] Ruis M.A.W., Te Brake J.H.A., Van de Burgwal J.A., De Jong I.C., Blokhuis H.J., Koolhaas J.M. (2000). Personalities in female domesticated pigs: Behavioral and physiological indications. Appl. Anim. Behav. Sci..

[25] NRC (2012). Nutrient Requirements of Swine.

[26] Altmann J. (1974). Observational Study of Behavior: Sampling Methods. Behaviour.

[27] Welfare Quality® (2009). Welfare Quality® Assessment Protocol for Pigs (Sows and Piglets, Growing and Finishing Pigs).

[28] Fels M., Hartung J., Hoy S. (2014). Social hierarchy formation in piglets mixed in different group compositions after weaning. Appl. Anim. Behav. Sci..

[29] Scheffler K., Stamer E., Traulsen I., Krieter J. (2016). Relationship between behavioural tests and agonistic interactions at different age levels in pigs. Appl. Anim. Behav. Sci..

[30] Langbein J., Puppe B. (2004). Analysing dominance relationships by sociometric methods—A plea for a more standardised and precise approach in farm animals. Appl. Anim. Behav. Sci..

[31] Samarakone T.S., Gonyou H.W. (2009). Domestic pigs alter their social strategy in response to social group size. Appl. Anim. Behav. Sci..

[32] McGlone J.J. (1985). A Quantitative Ethogram of Aggressive and Submissive Behaviors in Recently Regrouped Pigs. J Anim Sci..

[33] Dalmau A., Fabrega E., Velarde A. (2009). Fear assessment in pigs exposed to a novel object test. Appl. Anim. Behav. Sci..

[34] Colpoys J.D., Abell C.E., Gabler N.K., Keating A.F., Millman S.T., Siegford J.M., Young J.M., Johnson A.K. (2015). Feed efficiency effects on barrow and gilt behavioral reactivity to novel stimuli tests. J. Anim. Sci..

[35] Leliveld L.M.C., Düpjan S., Tuchscherer A., Puppe B. (2017). Vocal correlates of emotional reactivity within and across contexts in domestic pigs (Sus scrofa). Physiol. Behav..

[36] Appleby M.C. (1983). The probability of linearity in hierarchies. Anim. Behav..

[37] Heimburge S., Kanitz E., Otten W. (2019). The use of hair cortisol for the assessment of stress in animals. Gen. Comp. Endocrinol..

[38] Davenport M.D., Tiefenbacher S., Lutz C.K., Novaka M.A., Meyer J.S. (2006). Analysis of endogenous cortisol concentrations in the hair of rhesus macaques. Gen. Comp. Endocrinol..

[39] Abeni F., Petrera F., Prà A.D., Rapetti L., Crovetto G.M., Galassi G. (2018). Blood parameters in fattening pigs from two genetic types fed diet with three different protein concentrations. Transl. Anim. Sci..

[40] Thorn C.E., Bowman A.S., Eckersall D. (2022). Hematology of Pigs.

[41] Martínez-Miró S., Tecles F., Ramón M., Escribano D., Hernández F., Madrid J., Orengo J., Martínez-Subiela S., Manteca X., Cerón J.J. (2016). Causes, consequences and biomarkers of stress in swine: An update. BMC Vet. Res..

[42] Klem T.B., Bleken E., Morberg H., Thoresen S.I., Framstad T. (2010). Hematologic and biochemical reference intervals for Norwegian crossbreed grower pigs. Vet. Clin. Pathol..

[43] Montoro J.C., Pessoa J., Solà-Oriol D., Muns R., Gasa J., Manzanilla E.G. (2022). Effect of phase feeding, space allowance and mixing on productive performance of grower-finisher pigs. Animals.

[44] Foister S., Doeschl-Wilson A., Roehe R., Arnott G., Boyle L., Turner S. (2018). Social network properties predict chronic aggression in commercial pig systems. PLoS ONE.

[45] Coutellier L., Arnould C., Boissy A., Orgeur P. (2007). Pig’s responses to repeated social regrouping and relocation during the growing-finishing period. Appl. Anim. Behav. Sci..

[46] Désautés C., Bidanel J.P., Mormède P. (1997). Genetic study of behavioural and pituitary-adrenocortical reactivity in response to an environmental challenge in pigs. Physiol. Behav..

[47] Mormède P., Andanson S., Aupérin B., Beerda B., Guémené D., Malmkvist J., Manteca X., Manteuffel G., Prunet P., van Reenen C.G. (2007). Exploration of the hypothalamic-pituitary-adrenal function as a tool to evaluate animal welfare. Physiol. Behav..

[48] Ekkel E.D., Dieleman S.J., Schouten W.G.P., Portela A., Corni-Lissen G., Tielen M.J.M., Halberg F. (1996). The circadian rhythm of cortisol in the saliva of young pigs. Physiol. Behav..

[49] Ruis M.A.W., Brake J.H.A., Engel B., Ekkel E.D., Buist W.G., Blokhuis H.J., Koolhaas J.M. (1997). The circadian rhythm of salivary cortisol in growing pigs: Effects of age, gender, and stress. Physiol Behav..

[50] Leiber-Schotte C. (2009). Influence of the Ranking in Young Sires in the Self-Performance Test on Feed Intake and Feed Intake Behavior, Taking into Account Endocrinological and Immunological Parameters. Ph.D. thesis.

[51] Parois S., Larzul C., Prunier A. (2017). Associations between the dominance status and sexual development, skin lesions or feeding behaviour of intact male pigs. Appl. Anim. Behav. Sci..

[52] Bus J.D., Boumans I.J.M.M., Webb L.E., Bokkers E.A.M. (2021). The potential of feeding patterns to assess generic welfare in growing-finishing pigs. Appl. Anim. Behav. Sci..

[53] Morrow A.T.S., Walker N. (1994). A note on changes to feeding behaviour of growing pigs by fitting stalls to single-space feeders. Anim. Prod..

[54] Botermans J.A.M., Svendsen J. (2000). Effect of feeding environment on performance, injuries and behaviour in growing-finishing pigs: Group-based studies. Acta Agric. Scand. Sect. A—Anim. Sci..

[55] Andersen I.L., Andenæs H., Bøe K.E., Jensen P., Bakken M. (2000). The effects of weight asymmetry and resource distribution on aggression in groups of unacquainted pigs. Appl. Anim. Behav. Sci..

[56] McBride G., James J., Hodgens N. (1964). Social behaviour of domestic animals. IV. Growing pigs. Anim. Prod..

[57] Hafez E.S.E., Signoret J.P., Hafez E.S.E. (1969). The behaviour of swine. The Behaviour of Domestic Animals.

[58] Meese G.B., Ewbank R. (1972). A note on instability of the dominance hierarchy and variations in level of aggression within groups of fattening pigs. Anim. Prod..

[59] Koolhaas J.M., Van Reenen C.G. (2016). Interaction between coping style/personality, stress, and welfare: Relevance for domestic farm animals. Am. Soc. Anim. Sci..

[60] Finkemeier M.A., Langbein J., Puppe B. (2018). Personality research in mammalian farm animals: Concepts, measures, and relationship to welfare. Front. Vet. Sci..

[61] O’Malley C.I., Turner S.P., D’Eath R.B., Steibel J.P., Bates R.O., Ernst C.W., Siegford J.M. (2019). Animal personality in the management and welfare of pigs. Appl. Anim. Behav. Sci..

[62] Zebunke M., Nürnberg G., Melzer N., Puppe B. (2017). The backtest in pigs revisited Inter-situational behaviour and animal classification. Appl. Anim. Behav. Sci..

[63] Camerlink I., Ursinus W.W., Bartels A.C., Bijma P., Bolhuis J.E. (2018). Indirect genetic effects for growth in pigs affect behaviour and weight around weaning. Behav. Genet..

[64] Fraser D. (1974). The vocalizations and other behaviour of growing pigs in an ‘open field’ test. Appl. Anim. Ethol..

